# Primary refractory plasmablastic lymphoma: A precision oncology approach

**DOI:** 10.3389/fonc.2023.1129405

**Published:** 2023-02-27

**Authors:** Hanno M. Witte, Anke Fähnrich, Axel Künstner, Jörg Riedl, Stephanie M. J. Fliedner, Niklas Reimer, Nadine Hertel, Nikolas von Bubnoff, Veronica Bernard, Hartmut Merz, Hauke Busch, Alfred Feller, Niklas Gebauer

**Affiliations:** ^1^ Department of Hematology and Oncology, University Hospital of Schleswig-Holstein, Lübeck, Germany; ^2^ Department of Hematology and Oncology, Federal Armed Forces Hospital, Ulm, Germany; ^3^ Medical Systems Biology Group, Lübeck Institute of Experimental Dermatology, University of Lübeck, Lübeck, Germany; ^4^ Institute for Cardiogenetics, University of Lübeck, Lübeck, Germany; ^5^ University Cancer Center Schleswig-Holstein, University Hospital of Schleswig- Holstein, Lübeck, Germany; ^6^ Hämatopathologie Lübeck, Reference Centre for Lymph Node Pathology and Hematopathology, Lübeck, Germany

**Keywords:** molecular tumor board, plasmablastic lymphoma, whole exome sequencing, whole transcriptome sequencing, recurrent aberrations, targeted therapy

## Abstract

**Introduction:**

Hematologic malignancies are currently underrepresented in multidisciplinary molecular-tumor-boards (MTB). This study assesses the potential of precision-oncology in primary-refractory plasmablastic-lymphoma (prPBL), a highly lethal blood cancer.

**Methods:**

We evaluated clinicopathological and molecular-genetic data of 14 clinically annotated prPBL-patients from initial diagnosis. For this proof-of-concept study, we employed our certified institutional MTB-pipeline (University-Cancer-Center-Schleswig-Holstein, UCCSH) to annotate a comprehensive dataset within the scope of a virtual MTB-setting, ultimately recommending molecularly stratified therapies. Evidence-levels for MTB-recommendations were defined in accordance with the NCT/DKTK and ESCAT criteria.

**Results:**

Median age in the cohort was 76.5 years (range 56-91), 78.6% of patients were male, 50% were HIV-positive and clinical outcome was dismal. Comprehensive genomic/transcriptomic analysis revealed potential recommendations of a molecularly stratified treatment option with evidence-levels according to NCT/DKTK of at least m2B/ESCAT of at least IIIA were detected for all 14 prPBL-cases. In addition, immunohistochemical-assessment (CD19/CD30/CD38/CD79B) revealed targeted treatment-recommendations in all 14 cases. Genetic alterations were classified by treatment-baskets proposed by Horak et al. Hereby, we identified tyrosine-kinases (TK; n=4), PI3K-MTOR-AKT-pathway (PAM; n=3), cell-cycle-alterations (CC; n=2), RAF-MEK-ERK-cascade (RME; n=2), immune-evasion (IE; n=2), B-cell-targets (BCT; n=25) and others (OTH; n=4) for targeted treatment-recommendations. The minimum requirement for consideration of a drug within the scope of the study was FDA-fast-track development.

**Discussion:**

The presented proof-of-concept study demonstrates the clinical potential of precision-oncology, even in prPBL-patients. Due to the aggressive course of the disease, there is an urgent medical-need for personalized treatment approaches, and this population should be considered for MTB inclusion at the earliest time.

## Introduction

The success of targeted cancer therapies depends on the therapeutic detection of the targetable biomarker rather than the histologic subtype (1). Consequently, the number of basket trials investigating the efficacy of molecularly stratified therapeutic options is continuously increasing in recent years (2). Abandoning omnidirectional and unspecific treatment strategies, implementing multidisciplinary molecular tumor boards (MTB), and synchronous advancements in genomic profiling rapidly expand the spectrum of existing treatment strategies in cancer patients (3–5). To date, the implementation rate of effective MTB recommendations resulting in a beneficial outcome for cancer patients is in great need of improvement as most patients in the MTB setting are heavily pre-treated and at a very late stage within the course of the disease. Furthermore, the turn-around time for molecular and genetic diagnostic workups is between three and four weeks. Consecutively, recommended treatments are implemented in a minority of cases (4). Additionally, the performance of genomic profiling and allocation to rational therapies within the context of MTB evaluations is extremely heterogeneous (6, 7). Some Cancer Centers derive MTB recommendations merely from targeted panel sequencing, whereas others perform whole exome/genome sequencing (WES/WGS) potentially complemented with entire transcriptome sequencing (WTS) and epigenetic analyses resulting in a more refined understanding of variants and processes driving each cancer (6, 8, 9). However, the representation of hematologic malignancies in MTBs remains disproportionally low (4, 10) and MTB activities focus on solid tumors in most cases (4). Through implementing MTB platforms and growing experience with molecular diagnostics in a clinical setting, vast datasets for molecularly stratified treatments were generated (11). Consecutively, clinical outcome in personalized cancer therapies is steadily improving (12). At the same time, high-throughput sequencing and single-cell profiling allowed the refinement of the taxonomy, e.g. of aggressive B-cell non-Hodgkin lymphomas (B-NHL), uncovering novel potential therapeutically targetable vulnerabilities for personalized treatment strategies (13–17). Notably, the fusion of both fields, incorporating the advantages of state-of-the-art MTB diagnostics and decision-making as well as a growingly refined molecular understanding of hematological malignancies appear exceptionally promising, especially in rare entities associated with a dismal outcome such as plasmablastic lymphoma (PBL) (18). Poor prognosis and frequent concomitant HIV infections in younger PBL patients or immunodeficiency of other causes (e.g,. age-related immunosenescence or secondary to organ transplant recipients) underline the urgent need for novel therapeutic strategies (19, 20).

Moreover, this heterogeneous clientele of patients entails a relevant subgroup frequently not eligible for intensive treatment (21). The present study aimed to evaluate a subcohort of primary refractory (pr) PBL patients from a previous study by our group from the precision hematologist’s perspective applying the certified institutional MTB pipeline (University Cancer Center Schleswig-Holstein, UCCSH) to a highly lethal blood cancer (20). Based on the histopathological and immunophenotypic assessment, whole exome and transcriptome sequencing data, immunological and genetic targets were individually annotated within the scope of a virtual MTB setting. This resulted in recommending immunologically and/or molecularly stratified treatment strategies for prPBL patients.

## Methods

### Study design and patient characteristics

For this proof-of-concept study, a virtual MTB approach was conducted in a retrospectively assembled cohort of prPBL aiming to address an urgent unmet medical need in an extremely rare and aggressive subtype of B-cell non-Hodgkin lymphoma of post-germinal origin. As previously reported, screening of our institutional database revealed 76 PBL cases whose biopsy specimens were transferred to the reference center for Haematopathology, University Hospital Schleswig Holstein Campus Lübeck and Haematopathology Lübeck for centralized histopathologic expert review between January 1998 and December 2020. Due to the rarity of this entity, the sample size was not statistically predetermined. The number of cases included in the study corresponds to all PBL cases that have been referred to the reference center of Hematopathology within two decades. Investigations were not randomized, and investigators were not blinded. After excluding PBL cases with insufficient or unrepresentative tissue samples, the molecular landscape of PBL was characterized based on WES and WTS from 33 and 20 PBL cases, respectively (20). From this cohort, 14 PBL cases presented with the primary refractory disease were selected, deducing potential advantages from applying a certified MTB-pipeline approach in the era of precision oncology. No data were excluded from analyses.

### Genomic and transcriptomic analysis

**Figure 1:**
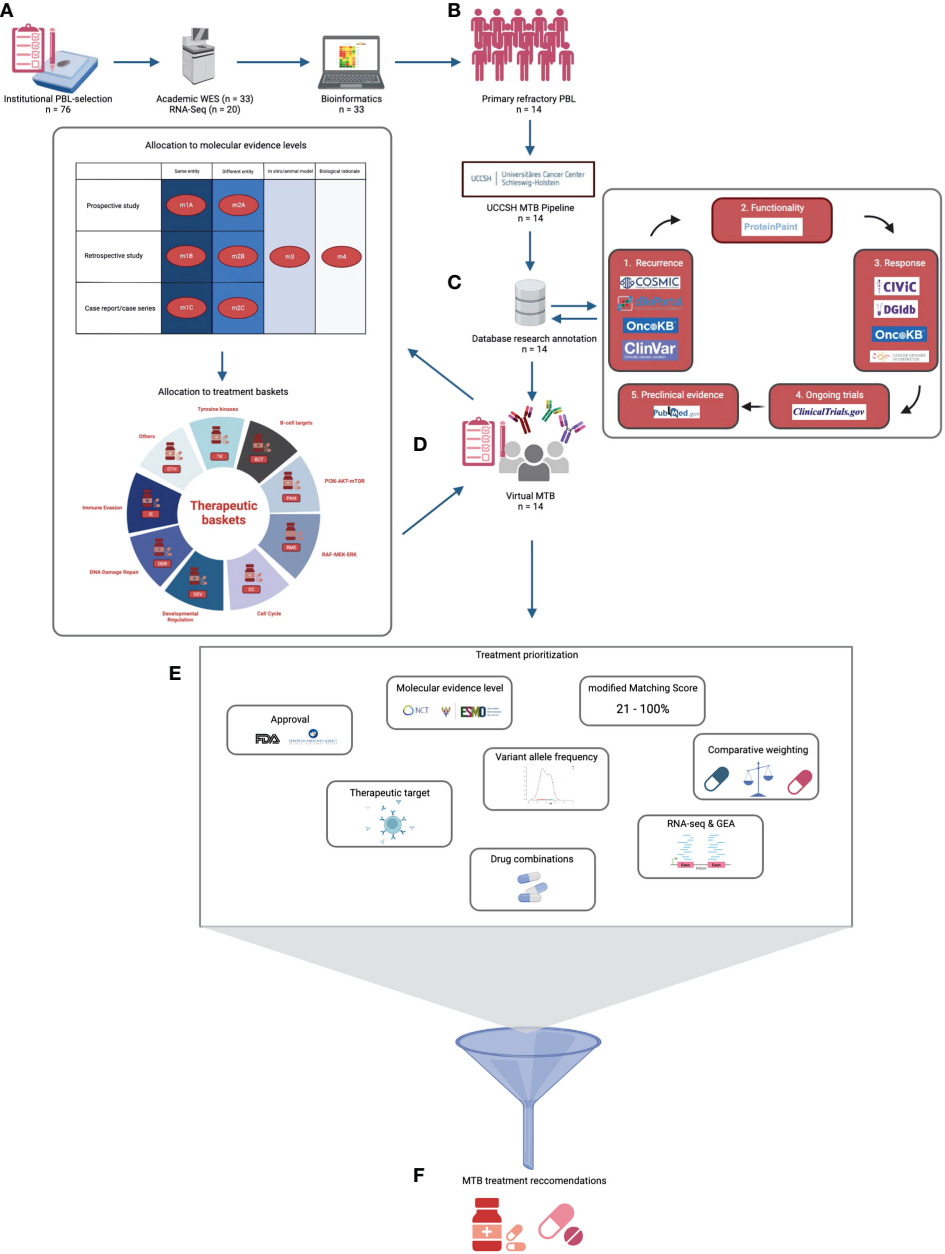
Virtual UCCSH molecular tumor board workflow for primary refractory plasmablastic lymphomas (prPBL). **(A)** After institutional PBL selection, academic WES and RNA-seq were performed in 33 and 20 cases, respectively. **(B)** Consecutively, 14 prPBL cases were identified and underwent UCCSH MTB pipeline evaluation. **(C)** Afterwards, manual database research annotation was conducted for each relevant variant. **(D)** In a virtual MTB setting, each prPBL case was discussed. Potential therapeutic vulnerabilities were allocated to molecular evidence levels and to treatment baskets. **(E)** Multifactorial treatment prioritization process revealed **(F)** MTB treatment recommendations.

Sample preparation, whole exome, and RNA-sequencing from formalin-fixed, paraffin-embedded (FFPE) tissue sections, as well as the process of raw data preparation, filtering, and the detection of single nucleotide variants (SNVs), short insertions and deletions (indels), somatic copy number aberrations (SCNAs) and fusion genes, were performed as previously described by Witte et al. (20) and Künstner et al. (22). MSI sensor was applied for the detection of microsatellite instability (MSI). For gene expression analysis from RNA-seq data, STAR ALIGNER (version 2.7.2b) and MIXNORM were used. The hg19 genome served as the reference genome. Several steps of bioinformatic analysis are integrated into the institutional MTB pipeline at University Cancer Center Schleswig-Holstein (UCCSH), which is certified for routine clinical diagnostics (**Figure 1**).

### MTB data preparation

Standardized sheets (genomic reports) that were constructed for MTB database research upon Medical Informatics for Research and Care in University Medicine (MIRACUM) pipeline analysis list the tumor mutational burden (TMB) and the percentual content of tumor cells (23). Apart from microsatellite (MSI) status, we calculated the *BRCA*ness score (cut-off ≥ 20%), and variant allele frequency (VAF) for each mutation and provided information regarding tumor heterogeneity as we reported on tumor subclones. Genomic reports were provided by the Medical Systems Biology Group (University of Lübeck). An individual genomic report was prepared for each case.

ANNOVAR was used for the annotation of filtered variants. Coverage for reference and alternative alleles for each variant was extracted using VCF- QUERY (VCFTOOLS version 0.1.13). Somatic variants were filtered as follows: at least 8 reads per base, minimum VAF of 5%, and only variants with a frequency below 0.001 in 1000 genomes, gnomAD, or ExAC, were considered for subsequent downstream analysis. Serving as a component for treatment prioritization, the effect of strong deleterious effects (CADD phred score > 20) was assessed per sample, and a gene set variation analysis was performed for WES and RNA-seq data against HALLMARK gene sets. More details on bioinformatics are provided in the **Supplementary Material**.

### MTB annotation and data analysis

In the first step, the recurrence of a genomic alteration was checked to employ the databases COSMIC, OncoKB (prognostic & diagnostic levels), ClinVar (clinically relevant variation), and cBioPortal. Afterward the functional relevance of an alteration was verified with ProteinPaint. Once an alteration was found to be recurrent as well as functionally relevant, its therapeutic vulnerability was annotated with CIVIC, OncoKB (therapeutic & FDA levels), Cancer-Genome-Interpreter (CGI), and the Drug-Gene-Interaction database (DGIdb) in a third step of database research (**Figure 1**). Apart from genomic alterations, we included immunohistochemical findings identified upon histopathologic diagnostics (CD19, CD30, CD38, and CD79B) to potentially serve as a relevant therapeutic target (**Supplemental Table 1**). A rationale for an immunotherapeutic strategy was recommended if a high TMB status was detected (≥10 mut/Mb) or in samples with MSI high status.

Finally, the annotation ended up concluding research of ongoing studies on https://clinicaltrials.gov and preclinical data on https://pubmed.ncbi.nlm.nih.gov. Only resources open for academic research purposes were considered for MTB recommendations. Evidence levels for MTB recommendations were defined in accordance with the NCT/DKTK MASTER program (**Figure 1**) and with the European Society for Medical Oncology Scale for Actionability of Molecular Targets (ESCAT) (4, 24). Additionally, genetic alterations were classified by biomarker/treatment baskets proposed by Horak et al. (**Figure 1**) (4).

### Prioritization of therapeutic vulnerabilities

The relevance of genomic alterations was considered based on VAF and CADD score. Variants of unknown significance were excluded. Each recommended drug was either approved by the Food and Drug Administration (FDA) and/or European Medicines Agency (EMA) or at least designated for FDA fast-track development as a minimum requirement for consideration within the scope of this study (cutoff date 31^st^ October 2022).

Treatment was recommended if at least one agent targeted at least one genomic alteration, one protein with relevant expression levels or if the TMB status was high. As data on the efficacy of agents targeting a non-mutated pathway component up- or downstream in an altered pathway detected upon gene set enrichment analysis remains insufficient, such agents were not considered for MTB recommendations. We excluded immunotherapeutic rationales based on the mutational signature SBS26, which was found in 6 cases as this rationale represents a biological rationale, so far lacking clinical validation (25).

Considering the matching score (MS) calculation by Sicklick et al., we adapted the calculation to our retrospective virtual MTB setting and provided a modified matching score (mMS) for each PBL case (26). The calculation was performed by dividing the number of alterations serving as a potential target for recommended drugs by the total number of characteristic alterations after excluding variants of unknown significance and a VAF lower than 5%. Apart from genomic alterations, immunohistochemical targets and immunotherapeutic rationales (TMB > 10mut/Mb, MSI status), as well as positive *BRCA*ness scoring pleading for PARP-inhibition, were considered equally for mMS calculation. In the present calculation, a synergistic and well-established drug combination targeting the same aberration (such as dabrafenib plus trametinib for *BRAF* mutations) the impact was counted as one. Results ranged from 0% to 100%. Higher scores represented better matches.


$$mMs(\%)=\frac{x}{y}\times 100$$

x = number of targetable vulnerabilities

(genomic alterations + IHC + MSI - high status + TMB - high status + BRCAness score)

y = number of characteristic and significant alterations

(targetable + un - targetable with a VAF ≥ 5%)

Combinations of drugs were considered and recommended according to the I-PREDICT study (9). Congruent to the approach of Sicklick et al. (9), the participating pharmacist screened each potential combination for feasibility in the light of drug interactions.

Each mutation was proved for its biological relevance and conclusiveness based on VAF, gene set enrichment analysis, and RNA-sequencing data, if available.

In summary, the prioritization of MTB recommendations was a multifactorial process simultaneously considering several considerations (**Figure 1**, **Supplementary Table 2**).

### Virtual MTB setting

The detailed MTB workflow is visualized in **Figure 1**. In total, we performed three rounds of virtual multicentre MTB conferences in accordance with the institutional standards of UCCSH, retrospectively discussing the 14 prPBL cases (1^st^ round: 4 cases; 2^nd^ round: 5 cases; 3^rd^ round: 5 cases). A board-certified hematologist presented the case. The conference included at least a molecular oncologist, a bioinformatician, a pathologist, a pharmacist, a radiologist, and a medical geneticist. Centralized documentation of MTB recommendations was conducted in each case (**Supplementary Material**).

### Data availability

Data was taken from accession number EGAD00001006795 (European genome-phenome archive (EGA)).

### Ethical regulation

This retrospective study was approved by the ethics committee of the University of Lübeck (reference no. 18-311), conducted in accordance with the Declaration of Helsinki, and patients have provided written informed consent regarding routine diagnostic and academic assessment, including genomic studies of their biopsy specimen in addition to the transfer of their clinical data.

## Results

### Patient characteristics and clinical outcome

Here, we report on the potential of molecularly stratified treatment options in 14 cases presenting with prPBL (median age 76.5 years, range 56 - 91). Additional PBL cases responding to initial cytoreductive treatment served as a comparison cohort (n = 19) (20). The majority of patients were male (11/14; 79%) and presented with advanced-stage disease (10/14; 71%) as well as an adverse prognostic constellation (11/14; 79% had an NCCN-IPI scoring ≥4). All patients had elevated lactate dehydrogenase (LDH) serum levels and the frequency of reduced performance status, according to the Eastern Cooperative Oncology Group (ECOG), demonstrated the frailty of patients included in the present cohort. Half of the current cohort was HIV positive (7/14 cases) and had underlying EBV (7/14 cases) infections at initial diagnosis. In 6 cases, we detected both HIV and EBV infections (43%). Cytoreductive treatment was applied in 13/14 cases (92.8%). More than half of the patients (8/14; 57%) received a CHOP-based (cyclophosphamide, hydroxydaunorubicin, vincristine, and prednisolone) treatment in 1^st^ line setting. In relapsed or refractory settings, 10/14 (71.4%) patients were eligible for 2^nd^ line cytoreductive treatment. Across any line of treatment, only five PBL patients responded to therapy (partial remission; PR), and another five PBL patients had stable disease (SD) as the best response. Three PBLs were completely refractory (progressive disease; PD) towards any treatment approach, and one PBL died shortly after initial clinical presentation. This underlines the urgent unmet clinical need for individualized treatment options among rare entities in hematology, such as PBL. Tumor cell content, immunohistochemical findings, and the contribution of *MYC* alterations were comparable between primary refractory cases and the comparison cohort. All baseline clinicopathological characteristics are summarized in **Table 1**.

**Table 1:** Baseline clinicopathological characteristics in primary refractory PBL.

Characteristic	Primary refractory PBL (n = 14)	Comparison cohort PBL (n = 19)
Age, median (range), y	76.5 (56 – 91)	60 (32 - 83)
Sex Female Male	3 (21%) 11 (79%)	7 (37%) 12 (63%)
HIV positivity	7 (50%)	7 (37%)
EBV positivity	7 (50%)	13 (68%)
HIV and EBV positivity	6 (43%)	10 (53%)
NCCN-IPI Low risk Low intermediate risk High intermediate risk High risk	- 3 (21%) 4 (29%) 7 (50%)	3 (16%) 7 (37%) 3 (16%) 6 (32%)
Stage (Ann Arbor) I - II III - IV	4 (29%) 10 (71%)	10 (53%) 9 (47%)
B-symptoms	8 (57%)	7 (37%)
Extranodal sites 0 1 - 2 >2	3 (21%) 10 (71%) 1 (7%)	1 (5%) 17 (89%) 1 (5%)
ECOG-PS 0 - 1 ≥ 2	4 (29%) 10 (71%)	12 (63%) 7 (37%)
Elevated LDH	14 (100%)	9 (47%)
Tumor cell content, median (range)	70% (60 - 90%)	70% (55 - 85%)
Immunohistochemistry CD38 CD19 CD30 CD79B Ki-67, median (range)	14 (100%) 5 (36%) 2 (14%) 4 (29%) 78% (60 - 90%)	19 (100%) 8 (42%) 5 (26%) 6 (32%) 80% (60 - 90%)
Chromosomal aberration *MYC* overall *MYC* amplification *MYC* split	10 (71%) 5 (36%) 5 (36%)	16 (84%) 7 (37%) 9 (47%)
Median TMB in mut/Mb (range)	4.06 (2.18 - 9.87)	3.09 (1.38 - 8.42)
Frontline therapy regimen CHOP-like Bendamustine-like Others Refusal or no treatment	8 (57%) 3 (21%) 2 (14%) 1 (7%)	13 (68%) - 4 (21%) 2 (11%)
Frontline therapy SAE (grade 3-5) Polyneuropathy Acute kidney injury Febrile neutropenia Sepsis	2 (14%) 3 (21%) 4 (29%) 5 (36%)	3 (16%) 2 (11%) 4 (21%) 2 (11%)

CHOP, cyclophosphamide/hydroxydaunorubicin/vincristine/prednisolone; EBV, Epstein Barr virus; ECOG-PS, Eastern Cooperative Oncology Group performance status; HIV, human immunodeficiency virus; LDH, lactate dehydrogenase; Mb, megabase; mut, mutations; NCCN-IPI, National Comprehensive Cancer Network International Prognostic Index; PBL, plasmablastic lymphoma; SAE, severe adverse event; TMB, tumor mutational burden; y, years.

**Figure 2:**
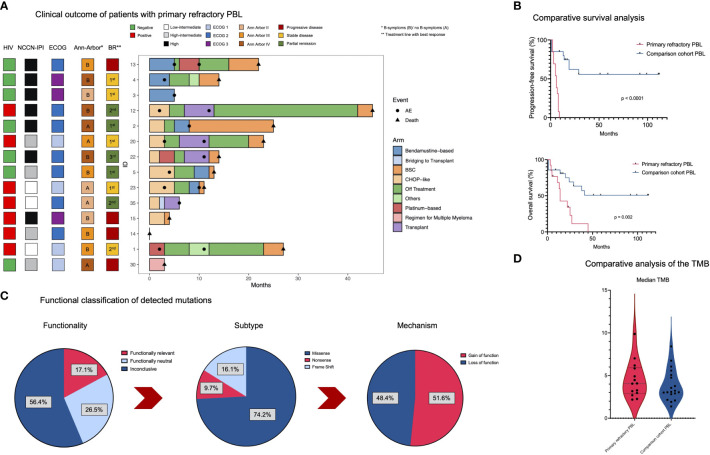
Clinical and genomic features in prPBL. **(A)** The swimmer plot illustrates the clinical course of the disease for each prPBL case. **(B)** Kaplan Meier survival analysis (PFS and OS) comparing prPBL cases and the comparison cohort which were not associated with primary refractory disease. **(C)** Pie charts outlining functionality, the mutational subtype and the mechanism of detected mutations. **(D)** Comparative median TMB calculation between prPBL and the comparison group.

The course of the disease, information on clinical characteristics, and treatment sequences for each prPBL case is visualized in **Figure 2A**. Survival analysis revealed significantly inferior PFS (p< 0.0001) and OS (p = 0.002) in prPBL compared to those cases responding to first-line treatment (**Figure 2B**).

### Genomic profiling in primary refractory plasmablastic lymphomas

Since the genomic and transcriptomic landscape of PBL recently has been described comprehensively (20, 27, 28), we carved out genomic features of primary refractory cases to detect potential therapeutic vulnerabilities in this difficult-to-treat hematologic malignancy and verified their biological significance based on transcriptomic data.

**Figure 3:**
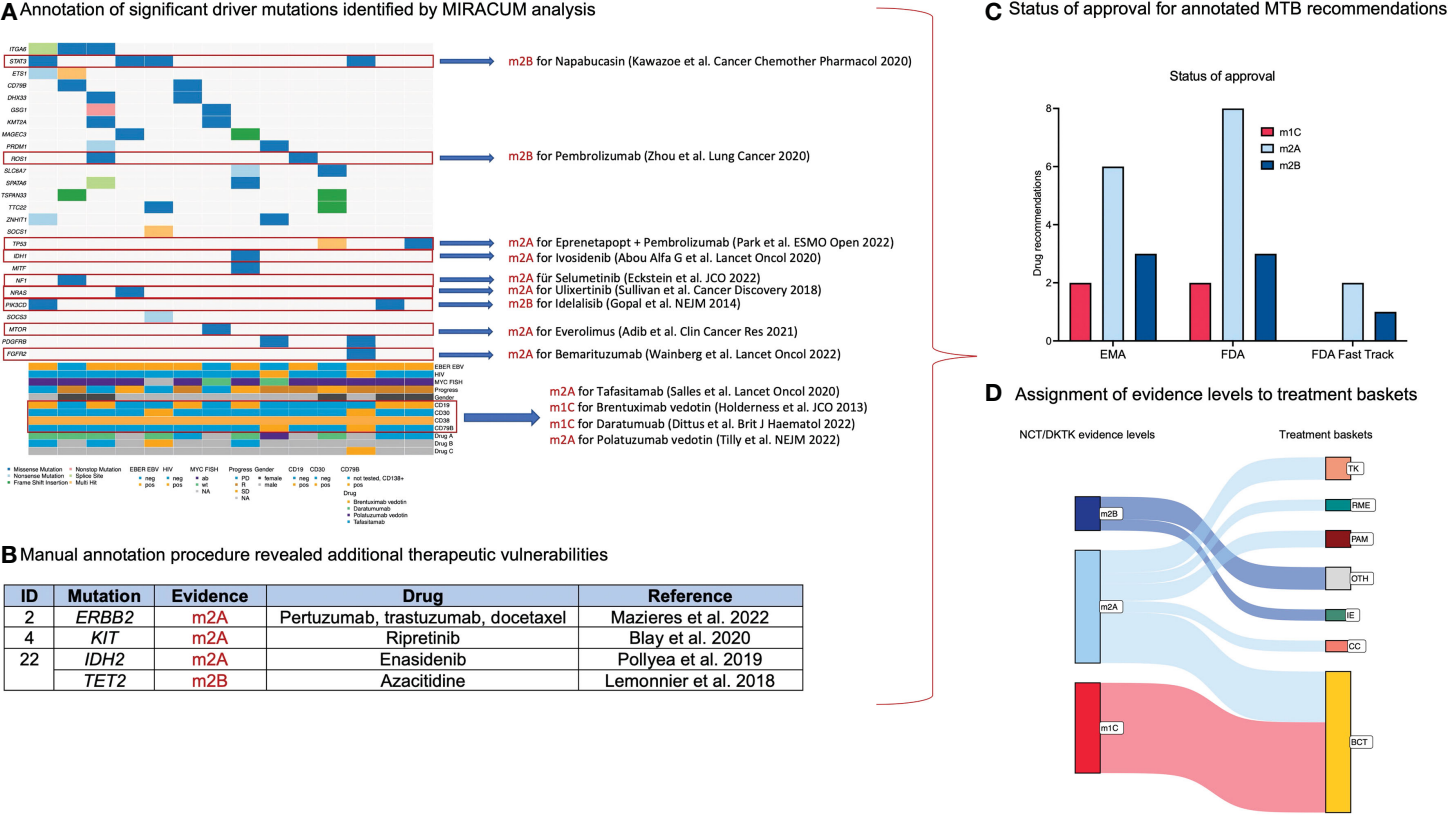
Results from manual database research and annotation of relevant genomic alterations. **(A)** Oncoplot summarizes relevant driver mutations and immunohistochemical targets detected upon MIRACUM pipeline analysis and potential therapeutic options. **(B)** Additional therapeutic vulnerabilities resulting from manual annotation procedure. **(C)** Bar plot visualizing the status of approval for annotated therapeutic options. **(D)** Sankey plot assigning molecular evidence levels to treatment baskets.

Our WES analysis revealed 3,955 SNVs and indels involving 2,700 genes after variant filtering. Investigations regarding the functionality of such mutations revealed that 17.1% were functionally relevant, whereas 26.5% were functionally neutral, and 56.4% were found to be functionally inconclusive. Across the detected mutations, the most frequent alterations were missense mutations (74.2%), followed by frameshift mutations (16.1%; indels) and nonsense mutations (9.7%). Further investigations revealed loss of function (LOF) in 48.4% and gain of function (GOF) in 51.6% among the spectrum of mutations (**Figure 2C**). The median TMB was slightly higher in primary refractory cases (4.06 mut/Mb in primary refractory PBL vs. 3.09 mut/Mb in the comparison cohort; **Figure 2D**; **Supplemental Table 3**). Given the limited sample size, this result was statistically insignificant (p = 0.337). TMB values in PBL displayed an overall low to intermediate TMB (29, 30). As previously reported, no evidence for MSI-based hypermutations in PBL, and matched germline DNA was not available for comparative analysis (20). After the identification of mutational subtypes and their functionality, the MIRACUM pipeline analysis identified 26 relevant genes carrying driver mutations (**Figure 3A**).

### Annotation of therapeutic vulnerabilities

In total, MIRACUM pipeline analysis identified 47 variants potentially driving cancer growth across 26 genes in the cohort of 14 prPBL. Among these variants, individualized database research assigned potential treatment recommendations for 15 variants involving 9 genes. For targets amenable to multiple agents, we favored agents with the highest level of evidence according to the NCT/DKTK classification (reference). The heterogeneous spectrum of treatment recommendations comprised well-known agents such as idelalisib (targeting *PI3KCD*) *(* 31) or everolimus (targeting *mTOR*) *(* 32) and novel agents such as eprenetapopt (targeting *TP53*) *(* 33), napabucasin (targeting *STAT3*) *(* 34) or bemarituzumab (targeting *FGFR2*) *(* 35). Moreover, recurrent LOF variants in *TP53* were previously related to treatment recommendations with CDK4/6 inhibitors (e.g., palbociclib/abemaciclib) (4). However, novel data suggest the inefficacy of CDK4/6 inhibitors in *TP53*-mutated malignancies as such mutations promote resistance to this class of drugs prompting us to exclude this recommendation (36). Results from the conducted MIRACUM pipeline analysis are outlined in **Figure 3A**.

Second, treatment recommendations based on immunohistochemical investigations were found in each case. Among the four immunohistochemical targets, a rationale for anti-CD38 (e.g., daratumumab) (37), anti-CD19 (e.g., tafasitamab) (38), anti-CD30 (brentuximab vedotin) (39) and anti-CD79B (e.g., polatuzumab vedotin) (40) was found in 14/14 (100%), 6/14 (43%), 2/14 (14%) and 2/14 (14%) cases, respectively (**Figure 3A**).

Third, exhaustive database research and annotation for recurrent genomic alterations beyond the MIRACUM pipeline analysis revealed four additional targetable mutations potentially acting as relevant drivers (*ERBB2, KIT, IDH2*, and *TET2*) (**Figure 3B**). In the era of precision oncology, alterations in such genes represent trailblazers for molecularly stratified treatment strategies. Further characteristics of annotated mutations are summarized in **Supplemental Table 4**. Particular attention was paid to the functional alignment concerning the biological significance and conclusiveness of each annotated mutation.

For potential treatment recommendations emerging from the annotation process, FDA approval was available for 14 agents, EMA was approved for 12 agents, and three agents were designated for FDA fast-track development (**Figure 3C**).

### Immunotherapeutic strategies and homologous recombination deficiency

There exists a rationale for immune checkpoint blockade in tumors with high TMB status and DNA MMR deficiency (41–44). We used a cut-off for TMB-high status of ≥10 mut/Mb (43). Contrary to expectations, all PBL cases presented with a TMB<10 mut/Mb. In six cases, we found a predominant DNA MMR deficiency signature (single base substitution signature SBS26) (25, 45). For this constellation, the rationale for immunotherapy based on a mutational signature associated with DNA MMR deficiency corresponds to a molecular evidence level no higher than m3/m4 (*in vitro* data/biologic rationale). Consequently, there was no TMB- or DNA-MMR-deficiency-related recommendation for immunotherapy in the present prPBL cohort. However, immune checkpoint blockade was recommended in two cases (X%) harboring *ROS1* alterations associated with resistance towards crizotinib and other targeted agents by propagating an immune escape mechanism (46). Moreover, we applied the calculation of the UCCSH MTB-pipeline BRCAness score (based on SBS6 signature) to predict the responsiveness towards PARP inhibitors (47). The *BRCA*ness score incorporates mutations coming along with homologous recombination deficiency (HRD), such as *BRCA1* or *BRCA2* losses or alterations mimicking these losses (*ATM, CHEK2, RAD51*) (48). In the present cohort, *BRCA*ness scoring revealed no evidence for PARP inhibition.

### Molecular evidence levels and assignment to therapeutic baskets

This proof-of-concept approach evaluated treatment options associated with NCT-DKTK molecular levels of evidence of at least m2B or higher for each prPBL case beyond anti-CD38 antibodies (daratumumab: 14x m1C rationale, ESCAT tier IIA; 32%). In the light of therapeutically addressable genomic alterations, 19 potential therapeutic vulnerabilities were assigned to m2A (n = 10; 23%) or m2B (n = 9; 20%) rationales. As already stated, immunohistochemical targets displayed a promising therapeutic option in primary refractory PBL. Assignment to molecular evidence levels revealed two m1C (brentuximab vedotin; 5%) and nine m2A (tafasitamab and polatuzumab vedotin; 20%) rationales.

Considering ESMO Scale for Clinical Actionability of molecular Targets (ESCAT) (reference), it has to be mentioned that recommendations based on higher ESCAT levels can hardly be reached in rare entities, especially among the spectrum of hematologic malignancies, due to the lack of prospective (randomized) clinical trials and their underrepresentation in MTB settings. Consequently, the experience in the molecularly stratified treatment of rare hematologic malignancies lags far behind recent developments in the field of solid tumors. However, the assignment of ESCAT evidence levels to therapeutic vulnerabilities found in this difficult-to-treat entity allocated 8 recommendations to ESCAT tier IC (18%), 14 recommendations to ESCAT tier IIA (32%), two recommendations to ESCAT tier IIB (5%) and 20 recommendations were allocated to ESCAT tier IIIA (45%). More preclinical options (NCT DKTK evidence level m3/m4 or ESCAT tier IV/V) were not considered in the present cohort, characterized by an urgent need for treatment recommendations in primary refractory setting due to the aggressive biologic features in PBL.

The calculation of the mMS ranged from 21% to 100% (median: 50%). In eight cases, a mMS of ≥ 50% was calculated (57%). The prognostic impact of the mMS remains speculative at this point, as no recommended treatment was administered in this virtual setting. However, the higher the mMS, the more alterations and pathways relevant to each case can be addressed upon recommended treatment strategies. Therefore, it can be expected that the mMS will be associated with overall response rates (ORR) and patient outcomes referring to the MS calculation reported in the I-PREDICT study (9).

**Figure 4:**
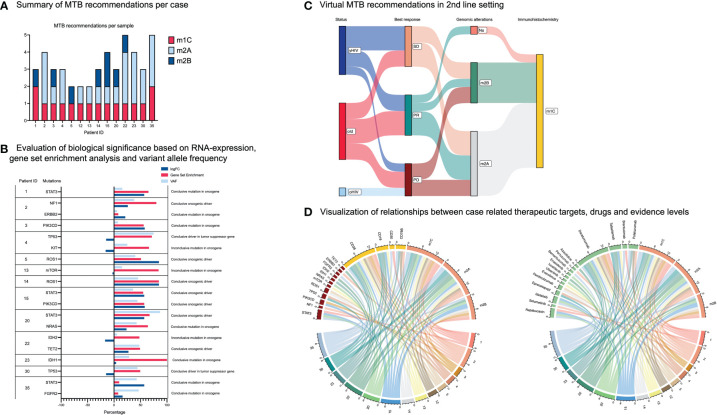
Therapeutic targets und treatment recommendations. **(A)** Case-related summary of MTB recommendations and associated molecular evidence levels. **(B)** Evaluation of RNA expression, gene set enrichment analysis and variant allele frequency (VAF) for each mutation serving as a therapeutic vulnerability. **(C)** Sankey plot allocating patients dependent on HIV status and age. The plot shows the best response of prPBL patients after standard chemotherapy. Moreover, the Sankey plot illustrates therapeutic vulnerabilities beyond standard chemotherapy based on genomic alterations and immunohistochemical targets. **(D)** Both chord plots demonstrate the relationships between cases and potential therapeutic targets, drugs and related evidence levels.

The assignment to treatment baskets was made under the consideration of the drug’s mechanism of action rather than its functionality regarding the targeted alteration. According to baskets, we identified tyrosine kinases (TK; n=5), PI3K-MTOR-AKT pathway (PAM; n=3), cell cycle alterations (CC; n=2), RAF-MEK-ERK cascade (RME; n=2), immune evasion (IE; n=2) and others (OTH; n=4) for targeted treatment recommendations. An additional treatment basket based on B-cell-specific immunohistochemical markers has been added: B-cell targets (BCT; n=25) representing the elementary therapeutic basket in the present study (**Figure 3D**). Our analysis revealed 3.0 MTB recommendations in the median across the entire cohort of prPBL (range 2 – 5) (**Figure 4A**). All potential MTB treatment recommendations are summarized in **Table 2**.

**Table 2:** Summary of MTB treatment recommendations.

ID	Target	Drug	NCT DKTK EL	ESCAT	Approval	mMS
1	CD38	Daratumumab	m1C	IIA	EMA/FDA	50%
	CD30	Brentuximab vedotin	m1C	IIB	EMA/FDA	
	STAT3	Napabucasin	m2B	IC	FDA-FT	
2	CD38	Daratumumab	m1C	IIA	EMA/FDA	44%
	CD79B	Polatuzumab vedotin	m2A	IIIA	EMA/FDA	
	NF1	Selumetinib	m2A	IIIA	EMA/FDA	
	ERBB2	Pertuzumab, trastuzumab, docetaxel	m2A	IIIA	EMA/FDA	
3	CD38	Daratumumab	m1C	IIA	EMA/FDA	50%
	PIK3CD	Idelalisib	m2B	IIIA	EMA/FDA	
4	CD38	Daratumumab	m1C	IIA	EMA/FDA	60%
	TP53	Eprenetapopt + pembrolizumab	m2A	IC	FDA-FT	
	KIT	Ripretinib	m2A	IIIA	EMA/FDA	
5	CD38	Daratumumab	m1C	IIA	EMA/FDA	43%
	ROS1	Pembrolizumab	m2B	IIIA	EMA/FDA	
12	CD38	Daratumumab	m1C	IIA	EMA/FDA	38%
	CD79B	Polatuzumab vedotin	m2A	IIIA	EMA/FDA	
13	CD38	Daratumumab	m1C	IIA	EMA/FDA	40%
	mTOR	Everolimus + pazopanib	m2A	IIIA	EMA/FDA	
14	CD38	Daratumumab	m1C	IIA	EMA/FDA	21%
	CD19	Tafasitamab	m2A	IIIA	EMA/FDA	
	ROS1	Pembrolizumab	m2B	IIIA	EMA/FDA	
15	CD38	Daratumumab	m1C	IIA	EMA/FDA	45%
	CD19	Tafasitamab	m2A	IIIA	EMA/FDA	
	STAT3	Napabucasin	m2B	IC	FDA-FT	
	PIK3CD	Idelalisib	m2B	IIIA	EMA/FDA	
20	CD38	Daratumumab	m1C	IIA	EMA/FDA	57%
	STAT3	Napabucasin	m2B	IC	FDA-FT	
	NRAS	Ulixertinib	m2A	IC	FDA-FT	
22	CD38	Daratumumab	m1C	IIA	EMA/FDA	50%
	CD19	Tafasitamab	m2A	IIIA	EMA/FDA	
	IDH2	Enasidenib + azacitidine	m2A	IIIA	FDA	
	TET2	Azacitidine	m2B	IIIA	EMA/FDA	
23	CD38	Daratumumab	m1C	IIA	EMA/FDA	57%
	CD19	Tafasitamab	m2A	IIIA	EMA/FDA	
	CD79B	Polatuzumab vedotin	m2A	IIIA	EMA/FDA	
	IDH1	Ivosidenib + azacitidine	m2A	IC	FDA	
30	CD38	Daratumumab	m1C	IIA	EMA/FDA	100%
	CD19	Tafasitamab	m2A	IIIA	EMA/FDA	
	TP53	Eprenetapopt + pembrolizumab	m2A	IC	FDA-FT	
35	CD38	Daratumumab	m1C	IIA	EMA/FDA	71%
	CD30	Brentuximab vedotin	m1C	IIB	EMA/FDA	
	CD79B	Polatuzumab vedotin	m2A	IIIA	EMA/FDA	
	STAT3	Napabucasin	m2B	IC	FDA-FT	
	FGFR2	Bemarituzumab	m2A	IIIA	EMA-FT	

### Transcriptomic profiling and verification of biological significance

RNA-sequencing data was available in 10 cases (71.4%). A gene set enrichment analysis and an integrated analysis of WES and RNA-seq data were performed for these cases. RNA expression patterns were used to underline or refute the biological significance of an annotated and potentially addressable mutation (**Figure 4B**). Based on this integrated analysis, one potential candidate driver mutation (*KIT*) was excluded as we evaluated inconclusive results for mRNA expression. *KIT* represents an oncogene, and consecutively we expected an overexpression of *KIT* mutation-related transcripts. However, the present analysis revealed an inconclusive under-expression. Others (n = 18) were found to be conclusive. We assumed driver mutations to be recurrent based on database research, functionally relevant based on RNA expression patterns, and represent an essential mutation within an altered pathway enriched in a sample (49). Following these criteria, we identified seven conclusive driver mutations (one in a tumor suppressor gene and six in oncogenes) and nine conclusive mutations in oncogenes, as well as one conclusive mutation in a tumor suppressor gene (**Figure 4B**). However, the significance of specific variants (driver or not) remained unresolved due to the lack of RNA-seq data in four cases. In such cases, a variant was categorized as a provisional mutation but not as a driver mutation.

Apart from this, RNA-seq did not reveal a distinct transcriptomic signature of prPBL compared to the comparison cohort (**Supplementary Figure 1**). However, we found exclusive expression patterns of *TAF9, CCDC125, ALMS1*and *ZNF462* in prPBL but not in the comparison cohort. RNA expression of such genes did not contribute novel insights into the pathogenesis of these difficult-to-treat cases.

Congruent to previous results published by our work group, the current re-evaluation of RNA-seq data did not reveal any novel, recurrent, and therapeutically relevant genomic fusion beyond those affecting *MYC* (**Supplemental Table 5**).

### Drug combinations

MTB recommendations for drug combinations considered insights from previous studies such as the I-PREDICT (NCT02534675) or the TOP-ART trials (NCT03127215) and other studies investigating the efficacy of drug combinations within the context of targeted therapies (9). Previous studies highlighted the advantages of drug combinations in molecularly stratified treatment settings (9). Preferably, drug combinations were chosen based on available datasets demonstrating their feasibility and efficacy in distinct entities or basket trials (11/14 cases; 79%). Novel drug combinations were selected considering the potential of overlapping drug toxicities, the molecular evidence levels for involved single agents, and the availability of such agents. Due to a distinct toxicity profile, immune checkpoint inhibition was a promising component for several drug combinations (4/14 cases; 29%). Moreover, several therapeutic options identified by immunohistochemical assessment harbored the potential for various additional drug combinations in all cases (**Supplemental Table 6**). There is growing evidence for using immunotherapeutic agents and/or agents targeting immunohistochemical assessable structures in the context of cytoreductive drug combinations in MTB settings as those combinations represent the standard of care among a variety of both solid and hematologic malignancies (40, 50, 51). However, there is still significant room for improvement in determining the combination of targeted therapeutics in the era of precision oncology.

### Summary of virtual MTB recommendations

The median turnaround time from DNA/RNA isolation to virtual MTB recommendations was 28 days. In this virtual MTB approach, solely recommendations on treatment but not additional diagnostics were enunciated. After the exclusion of one inconclusive mutation upon integrated WES and RNA-seq analysis, the standardized institutional UCCSH MTB pipeline application revealed 43 treatment recommendations across the 14 cases of prPBL. Because PBL represents an extremely rare and aggressive hematologic malignancy, most recommendations were based on evidence deduced from evidence generated in different entities (m2A-B; 28/43 recommendations; 65%). Subsequently, class m1 evidence (NCT-DKTK) was gained from case reports (m1C; 16/43 recommendations; 37%) as prospective clinical trials are in short supply (37, 39, 52).

As outlined in the methods section, treatment prioritization reflected a multifactorial process that incorporated patient-related clinical features (such as ECOG-PS), drug availability, molecular evidence levels, the calculation of the mMS, the modality of a therapeutic target, the biologic significance of an alteration (based on RNA-seq data, VAF and gene set enrichment analysis) and the feasibility of drug combinations. Most recommendations were based on single agents (38/43; 88%). The spectrum of MTB recommendations expanded when potential drug combinations were considered (28 additional recommendations, **Supplementary Table 6**), and/or alternative agents to preferred recommendations were considered as well (10 additional recommendations; **Supplementary Table 7**). The decision towards the preference for a specific agent over another addressing the same target was made based on available molecular evidence levels (e.g., daratumumab = m1C versus isatuximab = m2A).

In summary, whole exome and partially whole transcriptomic sequencing data of 14 primary refractory PBL cases were processed through the UCCSH MTB pipeline. They revealed a total of 43 treatment recommendations in this aggressive and chemo-refractory hematologic B-cell malignancy. Treatment recommendations comprised molecular evidence levels from m2B to m1C rationales. The heterogeneous distribution of treatment basket allocations underlines the diversity of potential treatment strategies in a virtual second-line setting (**Figure 4C**), and demonstrates the relevance of molecular diagnostics in rare and aggressive B-cell malignancies such as PBL. The interdependence between case-related targets, treatment recommendations, and evidence levels is visualized in **Figure 4D**.

## Discussion

Our virtual approach of a molecular tumor board provides evidence for promising therapeutic options and draws attention to an urgent medical need in this patient population which is yet underrepresented in MTB efforts. Including 1^st^ line treatment, therapeutic options are limited and often ineffective for PBL (53). Our work provides evidence that applying a validated MTB pipeline might open up therapeutic avenues for prPBL, a highly lethal blood cancer, serving as a role model for rare and aggressive hematologic neoplasms.

Several challenges are coming along with the introduction of an MTB process for patients with highly proliferative hematologic malignancies. One challenge is the transit of a histologic sample from making the correct diagnosis to MTB recommendations within a preferably short timeframe (54). Especially in highly aggressive malignancies, there is a relevant risk of terminal progression of the disease if molecularly targeted therapies are not rapidly identified and applied.

The PETAL trial demonstrated prognostic implications of interim fluorodeoxyglucose positron emission tomography (FDG-PET) imaging in patients with diffuse-large B-cell lymphoma (DLBCL) indicating poor event-free survival rates in patients with remaining PET-positivity after two cycles of R-CHOP-like immunochemotherapy (55). Outcome prediction by PET was independent of the International Prognostic Index (IPI) (55). This sparks the assumption that this observation might be transferred to other aggressive B-cell non-Hodgkin lymphomas such as PBL. Consecutively, PBL-cases with FDG-avidity upon interim PET-imaging may be associated with dismal prognosis and should be considered for early extened molecular testing. Therefore, we suggest including patients with rare, aggressive hematologic malignancies in clinical high-risk settings in precision oncology programs as early as possible, preferentially after the first evaluation of response in terms of interim FDG-PET (55). This would enable the identification of biomarkers for targeted therapeutic options in the relapsed or refractory setting at a point during the course of the disease when a successful bridging therapy may still be feasible. If previously identified biomarkers can be confirmed in the relapsed or refractory settings, this may accelerate the process of MTB treatment recommendations. Otherwise, novel targetable genomic alterations emerge in the relapsed or refractory setting, harboring novel options for targeted treatments (56). Moreover, repeated sampling might provide insights into the clonal evolution in such malignancies (57). This double-tracked strategy seems feasible in the light of cost efficacy, as financial analyses have shown that diagnostics in MTB settings represent 0.3% of total costs (58).

Such strategies require a simple and readily available diagnostic tool for the detection as well as monitoring of targetable genomic alterations over the course of disease. In recent years, liquid biopsy approaches analyzing cell-free DNA fragments (cfDNA)/circulating tumor DNA (ctDNA) from the peripheral blood have steadily evolved into an attractive component in genomic diagnostics (59). Liquid biopsies represent an extract of the current mutational status in a tumor, partially even reflecting subclonal architecture (60). To date, the essential critical aspects regarding the implementation in routine clinical use of liquid biopsy remains the insufficient sensitivity and lack of technological standardization between laboratories as well as pending results from prospective studies showing clinical benefit in a large scale prospective setting (61). Additionally, genomic profiling of tumor sites provides a more decisive overview on its molecular constitution leading to the most reliable identification of therapeutic vulnerabilities (62, 63). However, recent major technical advances have broadened the spectrum of molecular techniques leading to a more and more comprehensive convergence between the molecular studies from primary tumor tissues on the one hand and from the peripheral blood (liquid biopsy) on the other (64). We believe that there will be an essential role for liquid biopsy approaches in the upcoming era of precision oncology. Transferring the potential of liquid biopsy to our virtual MTB approach, we suggest its application from the initiation of a targeted and molecularly stratified treatment recommendation as a tool for drug monitoring, monitoring of response and for the detection of potential escape mechanisms related to the tumor (65, 66).

The lack of suitable basket trials for rare hematologic malignancies poses a significant challenge in applying molecularly stratified treatments. Consecutively, knowledge from MTB settings affecting solid tumors is often extrapolated into the field of hematology and generated molecular evidence, therefore, does hardly ever exceed the m2A level according to NCT/DKTK or ESCAT tier IIIA according to ESCAT recommendations. This also affects the transferability of established biomarkers associated with designated MTB rationales, such as olaparib therapy in malignancies with HRD deficiency or immune checkpoint inhibitor therapy in cancers harboring a DNA MMR mutational signature (24, 67). Moreover, there needs to be standardized practice to draw coherent conclusions from RNA-seq data and gene set enrichment analyses within an MTB setup. Accordingly, we used our RNA-seq data for an integrated analysis to verify the biological significance of mutations previously identified by WES. This integrated analysis supported the functional relevance for most mutations yet led to the exclusion of one modification (*KIT*) as a potential therapeutic target.

Including patients with rare hematologic malignancies in MTBs is associated with several chances. An essential finding of this study is that comprehensive genomic characterization of PBL revealed a broad range of promising therapeutically targetable vulnerabilities. Even today, many novel agents approved in the U.S. (FDA) and/or in Europe (EMA) are available and ready for clinical use, including a drug repurposing approach outside of the approved drug label. However, the steady increase of knowledge regarding the efficacy and toxicity profiles of novel agents in the light of molecularly stratified therapies used as single agents or as components in drug combinations will lead to a variety of therapeutic options in rare hematologic entities in which there is no supporting evidence beyond the application of CHO(E)P or an equivalent chemotherapeutic regimen with or without bortezomib (53, 68). Moreover, there is growing evidence for recommending drug combinations in MTBs (9). The stringent inclusion of rare hematologic malignancies into MTBs and basket trials will help gain experience regarding the application of drug combinations in such entities. Such processes require monitoring by Data Safety Monitoring Boards (9). Additionally, helpful tools such as matching score calculation should be used to predict the effectiveness and toxicity of single agents or drug combinations. In the present study, the calculation of the mMS is exclusively descriptive in nature. As this proof-of-concept study created a virtual MTB setting in which patients were not treated according to the recommended MTB strategies, the benefit of such strategy on the clinical outcome of PBL and other rare, aggressive hematologic neoplasms has yet to be demonstrated. In addition, further studies are needed to validate the modified way of MS calculation performed here and to define a practical cut-off value in a minimum p-value approach. The era of precision oncology is paralleled with the era of deep learning and machine learning approaches based on artificial intelligence (AI) models. Integrating AI into precision oncology is promising in order to standardize MTBs and to provide information on administered molecularly stratified therapies in a more standardized way (69). The increment of evidence in this rare entity associated with a high prevalence of HIV infections, is challenging as HIV infections pose a central exclusion criterion for the majority of clinical trials (70, 71). Currently, there exist negligible initiative on behalf of pharmaceutic companies regarding funding prospective clinical trials. Consecutively, the MTB setting represents a relevant alternative to gain more evidence in treating PBL and other rare, aggressive hematologic neoplasms.

Limitations of the present study predominantly include its limited sample size and shortcomings inherent to the retrospective nature harboring the potential of incomplete data, a selection bias during the inclusion procedure, and a detection bias during the analysis procedure, as well as limitations coming along with the fact that we present a solely virtual setting. Apart from WES, complete genomic RNA-seq data for each case and matched germline DNA for processing would have been desirable, including comparative analysis (4). In the present cohort, immunotherapeutic recommendations were concluded based on a mutational signature predominantly affecting DNA MMR genes. Immunohistochemical investigations included CD19, CD30, CD38, and CD79B but not programmed cell death ligand-1 (PD-L1) staining. PD-L1 staining probably extends the fraction of cases in which immune checkpoint blockade displays an appropriate therapeutic option, especially as a component of potential drug combinations.

In summary, the presented approach of an MTB for patients with prPBL is theoretical in nature. As none of the molecularly analyzed cases were treated according to the virtually recommended options in a prospective manner, optimal dosing as well as the applicability of considered drugs as monotherapy or as a combination needs further validation within the scope of clinical trials (e.g. umbrella and/or basket trials). A limited number of ongoing clinical trials will amplify the spectrum of therapeutic options in PBL and other rare hematologic malignancies investigating the efficacy of novel agents such as the anti-CD27 antibody varlilumab (NCT03038672) or the BCMA-directed antibody-drug conjugate belantamab mafodotin (NCT04676360).

## Conclusion

With the present study, we aim to draw attention to the potential benefits of a more frequent inclusion of rare hematologic malignancies such as PBL in MTB settings, as our work demonstrates the vast potential for molecularly stratified therapeutic approaches with reasonable molecular evidence levels. Such patients should therefore be introduced to precision oncology programs as early as possible due to the aggressive biology of the tumor. Our suggestion intends to initiate a learning process for improved patient care. To the best of our knowledge, this is the first approach of a virtual MTB in hematologic malignancies. Future studies are warranted to demonstrate the effectiveness and tolerability of molecularly stratified treatments in PBL patients and other rare hematologic neoplasms.

## Data availability statement

The datasets presented in this study can be found in online repositories. The names of the repository/repositories and accession number(s) can be found in the article.

## Ethics statement

The studies involving human participants were reviewed and approved by Ethics committee of the University of Lübeck (reference no. 18-311). The patients/participants provided their written informed consent to participate in this study. Written informed consent was obtained from the individual(s) for the publication of any potentially identifiable images or data included in this article.

## Author contributions

Study concept: HW, NG. Data collection: HW, NH, AF, AK, NR, VB, JR, HM, NB, HB, NG.

Data analysis and creation of figures and tables: HW, AF, AK, SF, NR, NB, HM, HB, AF, NG. Initial Draft of manuscript: HW. All authors contributed to the article and approved the submitted version.
