# Supplementary figures and images for: Primary refractory plasmablastic lymphoma: A precision oncology approach
Source: Front Oncol. 2023 Feb 27;13:1129405. doi: 10.3389/fonc.2023.1129405 (PMC10008852; doi:10.3389/fonc.2023.1129405)

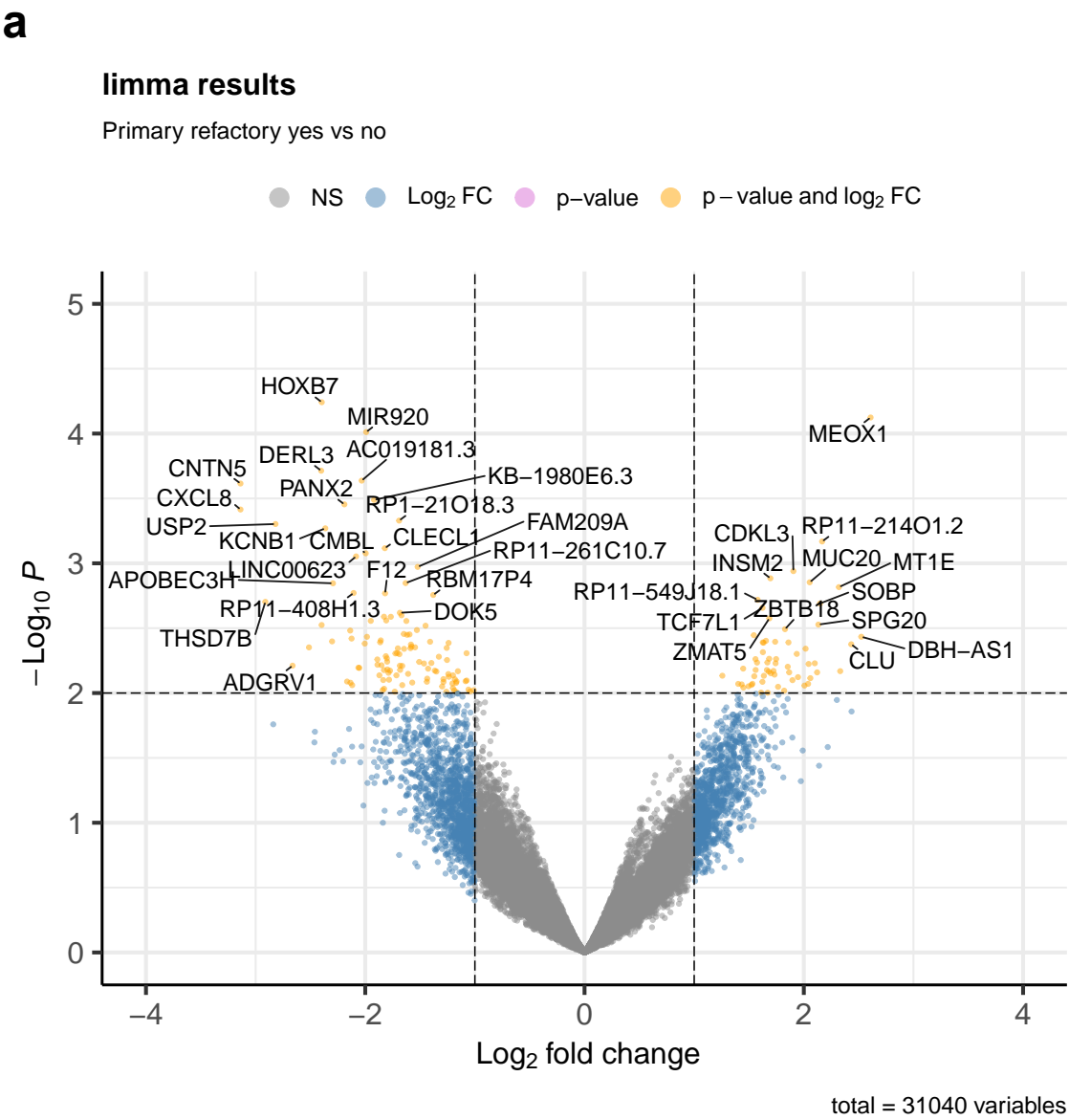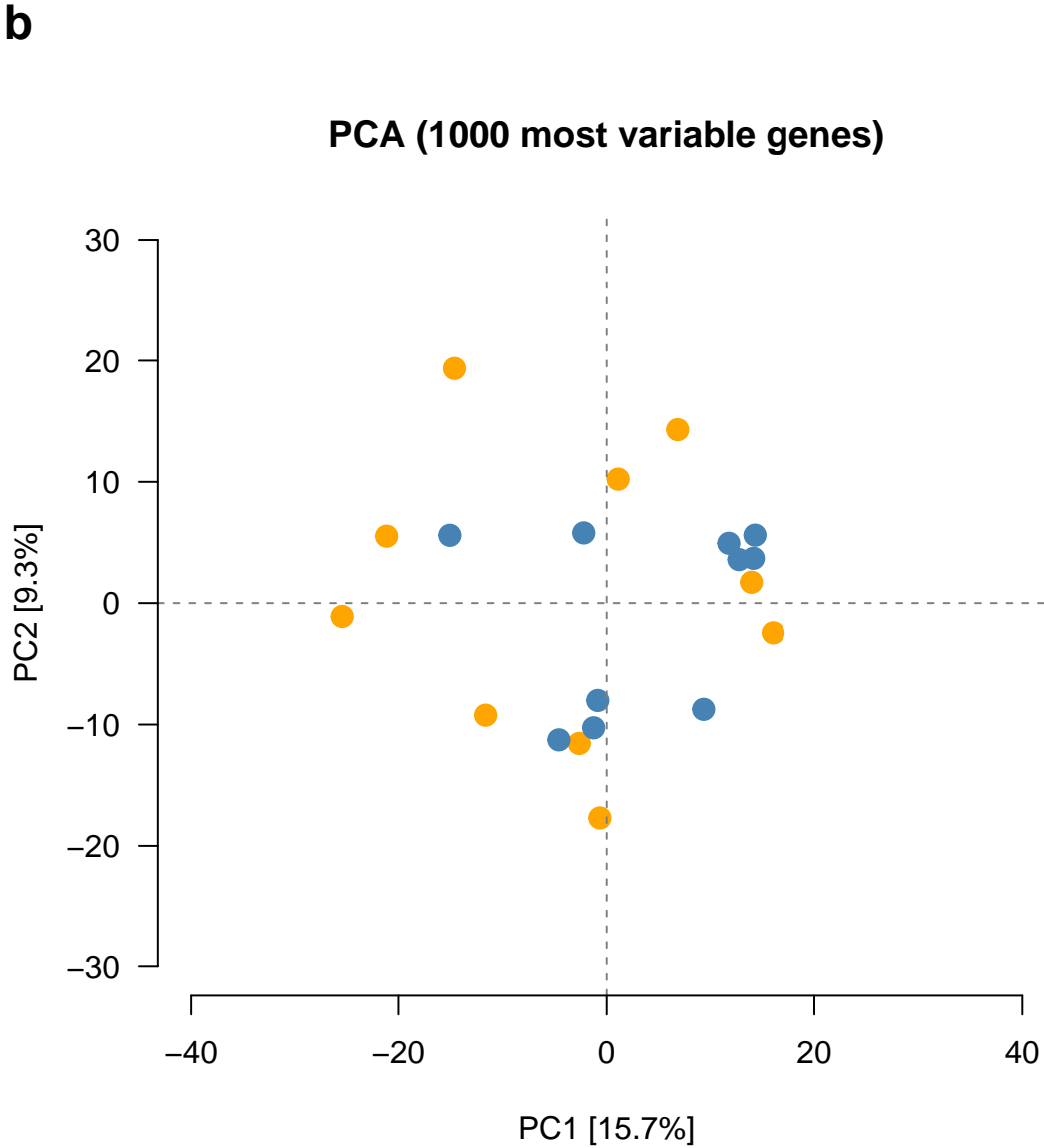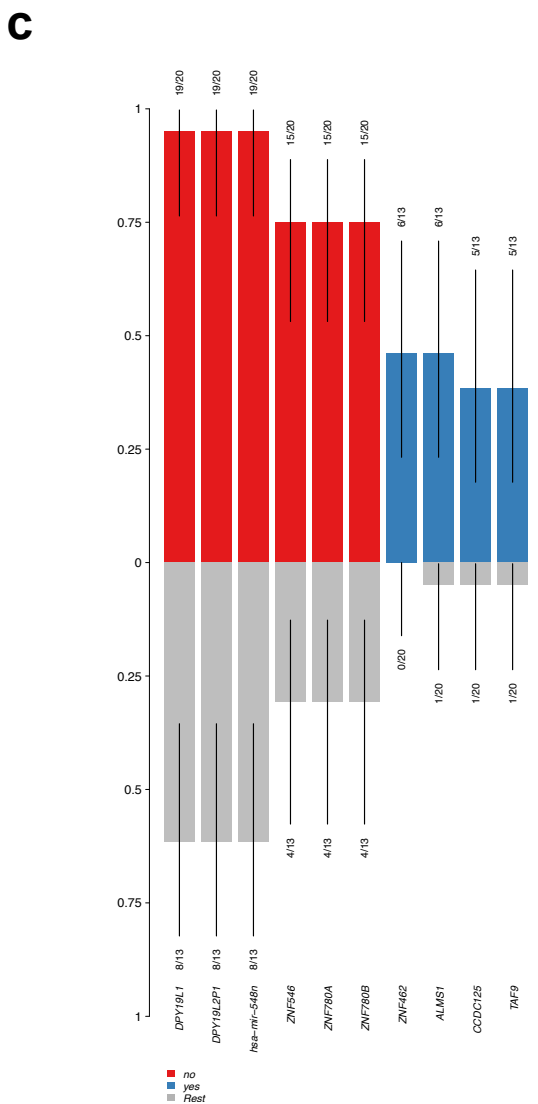

Supplement: Supplementary file 1 [file DataSheet_1.pdf]
