# Supplementary material for: Primary refractory plasmablastic lymphoma: A precision oncology approach
Source: Front Oncol. 2023 Feb 27;13:1129405. doi: 10.3389/fonc.2023.1129405 (PMC10008852; doi:10.3389/fonc.2023.1129405)
Supplement: Supplementary file 2 [file DataSheet_2.docx]

**Supplementary Material**

**Supplementary Methods**

*Whole exome and transcriptome sequencing*

As previously outlined in Witte *et al.,* genomic tumor DNA and tumor RNA were each extracted from 3 formalin-fixed, paraffin-embedded (FFPE) tissue sections of 5 mm thickness. WES was performed on a NovaSeq platform (Illumina). PBL samples were sequenced to an average depth of 201x (+/-50x; median, 203x). The investigation of the transcriptional profile was performed by RNA-seq. DNA (n = 33) and RNA (n = 20) sequencing were performed at Novogene (UK) Co., Ltd (1).

*Bioinformatic analysis*

The Medical Informatics in Research and Medicine (MIRACUM) pipeline was used to detect single nucleotide variants (SNVs), copy number variations (CNVs) as well as insertions and deletions (indels) from WES data. Additionally, tumor mutational burden (TMB), microsatellite instability (MSI) status and BRCAness were estimated for each sample by the MIRACUM pipeline as well.

ANNOVAR was used for annotation (e.g. CADD score, dann score, 1000 genomes/gnomAD/ExAC frequencies) of filtered variants. Somatic variants were filtered as follows: at least 8 reads per base, minimum VAF of 5%, and only variants with a frequency below 0.001 in 1000 genomes, gnomAD, or ExAC were considered for subsequent downstream analysis.

For gene expression analysis from RNA-seq data, STAR ALIGNER (version 2.7.2b) and MIXNORM were used. Additionally, fusion genes from RNA-seq data were identified by the application of STAR-FUSION (version 1.9.0) running in de novo reconstruction mode. The hg19 genome served as the reference genome. The validation of resulting fusion genes was performed by applying FUSIONCATCHER and FUSIONINSPECTOR.

*Ethical addendum*

All genomic studies were guided by the principles of the European Medicines Agency International Council for Harmonization of Technical Requirements for Registration of Pharmaceuticals for Human Use guideline E18 on genomic sampling and management of genomic data.

**Supplemental Figure Legend**

**Supplemental Figure 1.** Distinctive expression pattern between primary refractory PBL and PBL without primary progression (comparison cohort). The volcano plot (a) demonstrates distinct expressions without the affection of promising targetable vulnerabilities or prognostically characteristic and relevant genes. (b) shows results from the principal component analysis (PCA) illustrating the variance across the 1,000 most variable genes. (c) visualizes the detection of exclusive expression patterns in *TAF9, CCDC125, ALMS1*and *ZNF462* in prPBL but not in the comparison cohort.

**Supplemental Table 1**. Antibodies used.

| **Antibody** | **Supplier** | **Clone** | **Positivity cut-off** |
| --- | --- | --- | --- |
| CD38 | Leica Biosystems | SPC32 | - |
| CD19 | Dako | LE-CD19 | - |
| CD30 | Dako | BerH2 | 10% |
| CD79B | Dako | JCB117 | - |

**Supplemental Table 2.** Factors influencing prioritization process for MTB treatment recommendations.

| **Considerations for treatment prioritization process within the scope of MTB** |
| --- |
| - Status of approval (FDA/EMA/FDA fast track) |
| - Molecular evidence levels proposed by the NCT/DKTK MASTER program/ESCAT |
| - modified Matching score calculation |
| - The modality of a therapeutic target (driver mutation, IHC, MSI/TMB-high status) |
| - Targeting a relevant subset of tumor cells (the highest possible VAF) |
| - Biological significance based on gene set enrichment analysis and RNA-sequencing data |
| - Comparative weighting for recommended drugs based on efficacy data from literature |
| - Feasibility of drug combinations |
| DKTK, Deutsches Konsortium für Translationale Krebsforschung; EMA, European Medicines Agency; ESCAT, ESMO scale for clinical actionability of molecular targets; FDA, Food and Drug Administration; IHC, immunohistochemistry; MASTER, Molecularly Aided Stratification for Tumor Eradication; MSI, micro-satellite instability; MTB, molecular tumor board; NCT, nationales Zentrum für Tumorerkrankungen; RNA, ribonucleic acid; TMB, tumor mutational burden; VAF, variant allele frequency. |

**Supplemental Table 3.** DNA MMR/HRD signatures and TMB.

| **ID** | **DNA MMR** | **HRD** | **Mutational signature** | **TMB (mut/Mb)** |
| --- | --- | --- | --- | --- |
| 1 | - | 4.72% | SBS3 | 6.18 |
| 2 | 13.7% | - | SBS26 | 4.91 |
| 3 | - | - | - | 3.27 |
| 4 | - | - | - | 2.70 |
| 5 | 28.7% | 3.8% | SBS26; SBS3 | 4.05 |
| 12 | 17.9% | - | SBS26 | 3.17 |
| 13 | - | - | - | 7.01 |
| 14 | 15,1% | - | SBS26 | 9.87 |
| 15 | 11.7% | - | SBS26 | 4.47 |
| 20 | - | 7.88% | SBS3 | 2.96 |
| 22 | 18.2% | - | SBS26 | 5.84 |
| 23 | - |  | - | 1.92 |
| 30 | - | 11.8% | SBS3 | 4.08 |
| 35 | - | - | - | 2.26 |
| DNA MMR, deoxyribonucleic acid mismatch repair; HRD, homologous recombination deficiency; TMB, tumor mutational burden. | | | | |

**Supplemental Table 4.** Characteristics of annotated mutations.

| **ID** | **Gene** | **CADD Score** | **VAF** | **Tumor cell content** |
| --- | --- | --- | --- | --- |
| 1 | *STAT3* | 28.2 | 15.6% | 70% |
| 2 | *NF1* | - | 38.1% | 65% |
|  | *ERBB2* | - | 5.9% |  |
| 3 | *PIK3CD* | 29.4 | 7.3% | 70% |
| 4 | *TP53* | 23.7 | 74.8% | 60% |
|  | *KIT* | 22.9 | 25.0% |  |
| 5 | *ROS1* | - | 40.3% | 90% |
| 13 | *mTOR* | 24.2 | 15.0% | 65% |
| 14 | *ROS1* | 31.0 | 45.5% | 85% |
| 15 | *STAT3* | - | 36.0% | 70% |
|  | *PIK3CD* | - | 30.2% |  |
| 20 | *STAT3* | - | 86.9% | 80% |
|  | *NRAS* | - | 42.7% |  |
| 22 | *IDH2* | 24.2 | 5.7% | 85% |
|  | *TET2* | - | 48.1% |  |
| 23 | *IDH1* | - | 26.8% | 65% |
| 30 | *TP53* | 25.2 | 44.2% | 70% |
| 35 | *STAT3* | - | 42.8% | 60% |
|  | *FGFR2* | - | 46.7% |  |
| CADD, combined annotation dependent depletion; VAF, variant allele frequency. | | | | |

**Supplemental Table 5.** Fusion genes found in the prPBL cohort.

| **ID** | **Fusions** |
| --- | --- |
| 4 | MYC--IGH-@-ext |
| 23 | MSL2--GLCCI1 |
| 35 | TBL1XR1--EGFEM1P |
|  | IGH-@-ext--MYC |

**Supplemental Table 6.** Potential drug combinations.

| **ID** | **Drug combination** | **Targets** | **NCT/DKTK** | **References** |
| --- | --- | --- | --- | --- |
| 1 | Napabucasin + Pembrolizumab | *STAT3* | m4 | (2) |
|  | Daratumumab + ICE | *CD38* | m2C | (3) |
|  | Brentuximab + AVD | *CD30* | m2A | (4) |
| 2 | Pertuzumab + Trastuzumab + Docetaxel | *ERBB2* | m2A | (5) |
|  | Daratumumab + ICE | *CD38* | m2C | (3) |
| 3 | Idelalisib + Bendamustine | *PIK3CD* | m4 | (6) |
|  | Daratumumab + ICE | *CD38* | m2C | (3) |
| 4 | Eprenetapopt + Pembrolizumab | *TP53* | m2A | (7) |
|  | Daratumumab + ICE | *CD38* | m2C | (3) |
| 5 | Daratumumab + Pembrolizumab | *CD38 + ROS1* | m2A | (8) |
|  | Daratumumab + ICE | *CD38* | m2C | (3) |
| 12 | Daratumumab + ICE | *CD38* | m2C | (3) |
| 13 | Everolimus + Pazopanib | *mTOR* | m2A | (9) |
|  | Daratumumab + ICE | *CD38* | m2C | (3) |
| 14 | Daratumumab + Pembrolizumab | *CD38 + ROS1* | m2A | (8) |
|  | Daratumumab + ICE | *CD38* | m2C | (3) |
|  | Tafasitamab + Lenalidomide | *CD19* | m2A | (10) |
| 15 | Napabucasin + Pembrolizumab | *STAT3* | m4 | (2) |
|  | Daratumumab + ICE | *CD38* | m2C | (3) |
|  | Tafasitamab + Lenalidomide | *CD19* | m2A | (10) |
| 20 | Ulixertinib + Palbociclib | *NRAS* | m3 | (11) |
|  | Daratumumab + ICE | *CD38* | m2C | (3) |
| 22 | Enasidenib + Azacitidine | *IDH2* | m2A | (12) |
|  | Daratumumab + ICE | *CD38* | m2C | (3) |
|  | Tafasitamab + Lenalidomide | *CD19* | m2A | (10) |
| 23 | Ivosidenib + Azacitidine | *IDH1* | m2A | (13) |
|  | Daratumumab + ICE | *CD38* | m2C | (3) |
|  | Tafasitamab + Lenalidomide | *CD19* | m2A | (10) |
| 30 | Eprenetapopt + Pembrolizumab | *TP53* | m2A | (7) |
|  | Daratumumab + ICE | *CD38* | m2C | (3) |
|  | Tafasitamab + Lenalidomide | *CD19* | m2A | (10) |
| 35 | Napabucasin + Pembrolizumab | *STAT3* | m4 | (2) |
|  | Daratumumab + ICE | *CD38* | m2C | (3) |
|  | Brentuximab + AVD | *CD30* | m2A | (4) |
| DKTK, Deutsches Konsortium für Translationale Krebsforschung; NCT, Nationales Zentrum für Tumorerkrankungen | | | | |

**Supplementary Table 7.** Alternative agents for targets identified upon UCCSH MTB pipeline application.

| **Target** | **Alternative drug** | **NCT DKTK EL** |
| --- | --- | --- |
| CD38 | Isatuximab | m2A |
| CD19 | Blinatumumab | m2A |
| CD19 | Axicabtagene ciloleucel (CAR-T-cells) | m2A |
| ERBB2 | Trastuzumab deruxtecan | m2A |
| KIT | Imatinib | m2A |
|  | Sunitinib | m2A |
|  | Sorafenib | m2A |
| mTOR | Sirolimus | m2B |
|  | Temsirolimus | m2B |
| IDH1 | Olutasidenib | m2A |
| DKTK, Deutsches Konsortium für Translationale Krebsforschung; NCT, Nationales Zentrum für Tumorerkrankungen | | |

**Supplementary References**

1. Witte HM, Kunstner A, Hertel N, et al. Integrative genomic and transcriptomic analysis in plasmablastic lymphoma identifies disruption of key regulatory pathways. Blood Adv. 2022;6(2):637-651.

2. Kawazoe A, Kuboki Y, Shinozaki E, et al. Multicenter Phase I/II Trial of Napabucasin and Pembrolizumab in Patients with Metastatic Colorectal Cancer (EPOC1503/SCOOP Trial). Clin Cancer Res. 2020;26(22):5887-5894.

3. Dittus C, Miller JA, Wehbie R, Castillo JJ. Daratumumab with ifosfamide, carboplatin and etoposide for the treatment of relapsed plasmablastic lymphoma. Br J Haematol. 2022;198(2):e32-e34.

4. Connors JM, Radford JA. Brentuximab Vedotin for Stage III or IV Hodgkin's Lymphoma. N Engl J Med. 2018;378(16):1560-1561.

5. Mazieres J, Lafitte C, Ricordel C, et al. Combination of Trastuzumab, Pertuzumab, and Docetaxel in Patients With Advanced Non-Small-Cell Lung Cancer Harboring HER2 Mutations: Results From the IFCT-1703 R2D2 Trial. J Clin Oncol. 2022;40(7):719-728.

6. de Vos S, Wagner-Johnston ND, Coutre SE, et al. Combinations of idelalisib with rituximab and/or bendamustine in patients with recurrent indolent non-Hodgkin lymphoma. Blood Adv. 2016;1(2):122-131.

7. Park H, Shapiro GI, Gao X, et al. Phase Ib study of eprenetapopt (APR-246) in combination with pembrolizumab in patients with advanced or metastatic solid tumors. ESMO Open. 2022;7(5):100573.

8. Paul B, Symanowski J, Osipoff P, et al. A Phase 2 Trial of Daratumumab and Pembrolizumab in Refractory Multiple Myeloma. Blood. 2020;136(Supplement 1):2-2.

9. Bellmunt J, Lalani AA, Jacobus S, et al. Everolimus and pazopanib (E/P) benefit genomically selected patients with metastatic urothelial carcinoma. Br J Cancer. 2018;119(6):707-712.

10. Salles G, Duell J, Gonzalez Barca E, et al. Tafasitamab plus lenalidomide in relapsed or refractory diffuse large B-cell lymphoma (L-MIND): a multicentre, prospective, single-arm, phase 2 study. Lancet Oncol. 2020;21(7):978-988.

11. Emery CM, Corgiat B, Davis J, et al. Abstract 1057: Significant efficacy demonstrated with the combination of ulixertinib (ERK1/2 inhibitor) and CDK4/6 inhibitors in MAPK altered models. Cancer Research. 2022;82(12_Supplement):1057-1057.

12. DiNardo CD, Schuh AC, Stein EM, et al. Enasidenib plus azacitidine versus azacitidine alone in patients with newly diagnosed, mutant-IDH2 acute myeloid leukaemia (AG221-AML-005): a single-arm, phase 1b and randomised, phase 2 trial. Lancet Oncol. 2021;22(11):1597-1608.

13. Montesinos P, Recher C, Vives S, et al. Ivosidenib and Azacitidine in IDH1-Mutated Acute Myeloid Leukemia. N Engl J Med. 2022;386(16):1519-1531.
